# Effect of gravity on brain structure as indicated on upright computed tomography

**DOI:** 10.1038/s41598-020-79695-z

**Published:** 2021-01-11

**Authors:** Yoichi Yokoyama, Yoshitake Yamada, Kenzo Kosugi, Minoru Yamada, Keiichi Narita, Takehiro Nakahara, Hirokazu Fujiwara, Masahiro Toda, Masahiro Jinzaki

**Affiliations:** 1 Department of Radiology, Keio University School of Medicine, 35 Shinanomachi, Shinjuku-ku, Tokyo, 160-8582 Japan; 2 Department of Neurosurgery, Keio University School of Medicine, 35 Shinanomachi, Shinjuku-ku, Tokyo, 160-8582 Japan

**Keywords:** Anatomy, Neurology

## Abstract

We aimed to use upright computed tomography (CT) to depict posture-related changes in the brain tissue under normal gravity. Thirty-two asymptomatic volunteers underwent upright CT in the sitting position and conventional CT in the supine position on the same day. We compared the shift of the pineal body, cerebellar tonsil, the length of pituitary stalk, optic nerve sheath area and perimeter (ONSA and ONSP, respectively), and lateral ventricular volume between the supine and sitting positions. We also compared shape changes of the cerebrospinal fluid (CSF) spaces at different sites between both positions. In the sitting position, the pineal body shifted 0.68 ± 0.27 mm in the ventral direction and 0.76 ± 0.24 mm in the caudal direction, the length of pituitary stalk decreased by 1.23 ± 0.71 mm, the cerebellar tonsil descended by 2.10 ± 0.86 mm, the right ONSA decreased by 15.21 ± 6.54%, the left ONSA decreased by 15.30 ± 7.37%, the right ONSP decreased by 8.52 ± 3.91%, the left ONSP decreased by 8.20 ± 4.38%, and the lateral ventricular volume decreased by 5.07 ± 3.24% (all P < 0.001). We also observed changes in the shape of CSF spaces with changes in posture. We concluded that the intracranial structure of healthy subjects and volume of ventricles changed according to posture on Earth.

## Introduction

The intracranial structure is not known to change in the supine or sitting positions under normal (= 1 G) gravity. In a previous vertical magnetic resonance imaging (MRI) study, the intracranial structure was found to be highly conserved, both with respect to the skull and among individual cerebral domains^1^. Moreover, the gravitational effect on brain structures was negligible; there was no significant change in the cerebellar tonsillar position between the upright and supine positions in normal subjects^2^.

However, recently, it was reported that microgravity in outer space results in an upward shift of the brain and narrowing of cerebrospinal fluid (CSF) spaces after long-duration space flights^3^. As the brain shifts upward, the optic nerve is assumed to be pulled rearward and enlarged in the optic nerve sheath diameter (ONSD)^4^. This phenomenon will cause space flight-associated neuro-ocular syndrome (SANS), which the National Aeronautics and Space Administration has advocated^5^. An upright computed tomography (CT) scanner, whose physical characteristics are comparable to those of a conventional CT machine, has recently been developed^6^. The usefulness of upright CT has been already shown in other studies^6–8^. This CT scanner can acquire 0.5-mm iso-voxel data^6^, which are much thinner slice thicknesses than the 4.0–5.0-mm slice thicknesses obtained in previous MRI studies^1,2^.

On the basis of these recent findings and technical developments, we postulated that the intracranial structure would show slight positional change under Earth’s gravity, which has not been detected by the slice thicknesses of MRI. The purpose of this study was to evaluate the posture-based changes in the intracranial structure under normal gravity using the newly developed upright CT.

## Methods

### Study population

This study was conducted in Keio University Hospital, after institutional review board approval (trial registry number: 20160384) was granted and written informed consent was obtained from all the participants. All methods were carried out in accordance with relevant guidelines and regulations. Data were prospectively collected between April 2018 and October 2018. Thirty-two consecutive volunteers with no symptoms from a volunteer recruitment company, consisting of 16 men and 16 women (4 men and 4 women from each of the 30-, 40-, 50-, and 60-year age groups), were enrolled (Table 1). In order to assess the normal brain anatomy, subjects with a history of smoking, diabetes, hypertension, awareness of dysuria, and those who had previously undergone an operation or were undergoing treatment at the time, were excluded. These 32 subjects were analysed for normalcy of the great vessels and pelvic floor in a previous study^6^.

**Table 1:** Characteristics of the study population.

Characteristics of the study population (*n* = 32)			
	Male (n = 16)	Female (n = 16)	*P-*value
Age (years)	48.3 ± 13.0	48.4 ± 10.2	0.987
Body weight (kg)	65.9 ± 10.8	54.7 ± 7.50	< 0.001
Height (cm)	168.1 ± 6.3	158.6 ± 6.0	0.001
BMI (kg/m^2^)	23.3 ± 3.36	21.7 ± 2.38	0.059

*BMI*: body mass index.

### Image acquisition

All subjects prospectively underwent both upright CT (TSX-401R, Canon Medical Systems, Canon Medical Systems, Otawara, Japan) in the sitting position and conventional 320-detector row CT (Aquilion ONE, Canon Medical Systems, Otawara, Japan) in the supine position within two hours on the same day in the evening. Of the 32 volunteers, 30 were assessed in the supine position first, followed by the sitting position; the remaining 2 volunteers underwent the opposite protocol.

**Figure 1:**
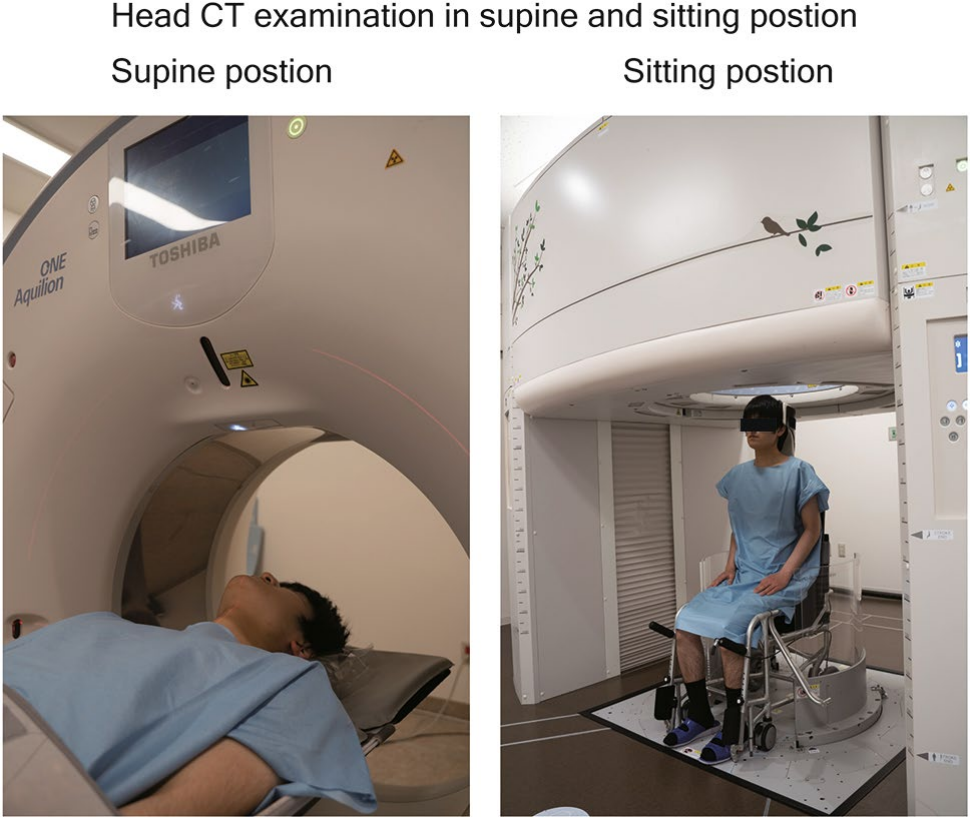
The CT examination in supine (left image) and sitting position (right image). In both the upright and conventional CT scanner, the subjects were instructed to keep their back straightened and look straight forward. Note: We have received informed consent for publication from the volunteer to have the face of the volunteer appear in the figure.

In both the upright and conventional CT scanner, the subjects were instructed to keep their back straightened and look straight ahead (Fig. 1: We have received informed consent for publication from the volunteer to have the face of the volunteer appear in the figure.). Scanning in all subjects was performed at 120 kVp, with 0.5 s of gantry rotation, in helical scan mode (80-row detector), with a noise index of 4 for 5-mm slice thickness, a helical pitch of 0.64, 512 × 512 matrix, and field of view of 320 mm. CT images with 0.5-mm cross-sectional thickness and 0.5-mm cross-section intervals were obtained.

### Measurements

All parameters were measured in a commercially available workstation, Synapse Vincent (FUJIFILM Medical Co., Ltd., Lexington, MA, USA). Each supine and sitting image underwent rigid registration; we subtracted the supine images from the registered sitting image. We confirmed that all the bone structures were subtracted, which indicates that rigid registration of the bone had been conducted accurately (SI 1). We measured the shift of the pineal body, length of the pituitary stalk, distance between the cerebellar tonsil and the BO line, ONSA, ONSP, and lateral ventricular volume.

**Figure 2:**
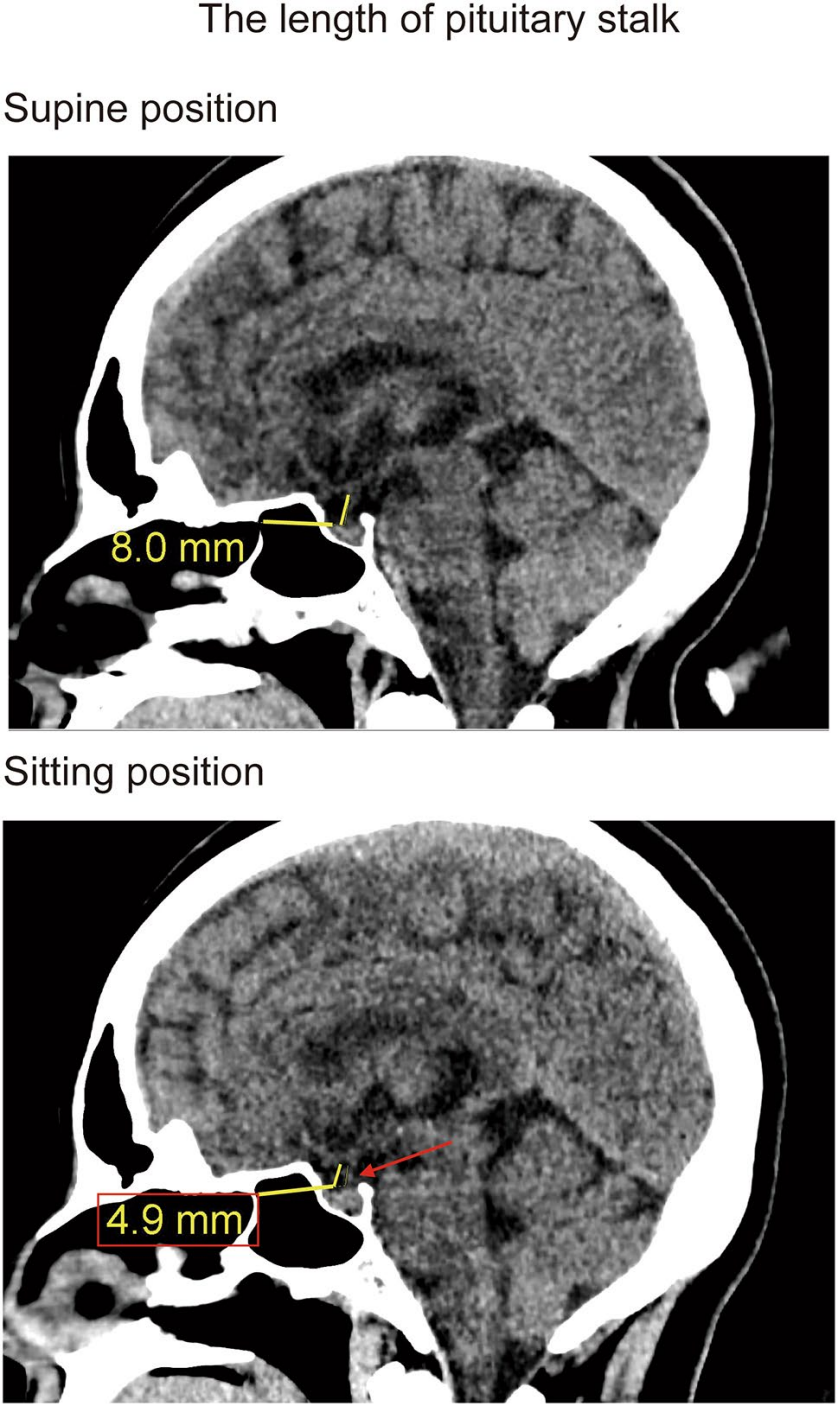
The length of pituitary stalk in supine (upper image) and sitting position (under image). The pituitary stalk shortened in sitting position (red arrow in sitting position).

**Figure 3:**
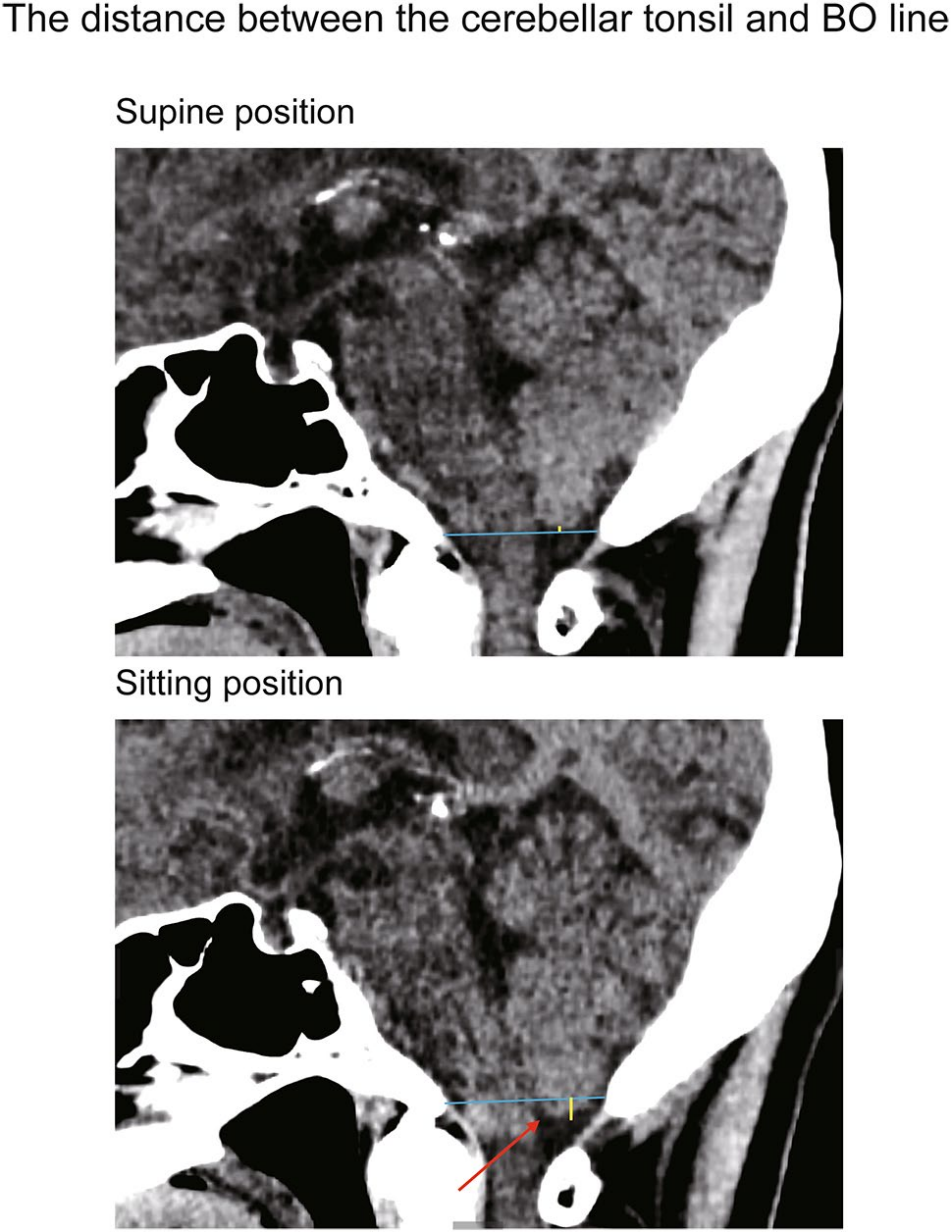
The distance between the cerebellar tonsil and BO line in supine (upper image) and sitting position (under image). The distance between the cerebellar tonsil and BO line shows yellow line, and BO line shows the blue line in picture. The tip of cerebellar tonsil was descended in sitting position (red arrow in sitting position).

**Figure 4:**
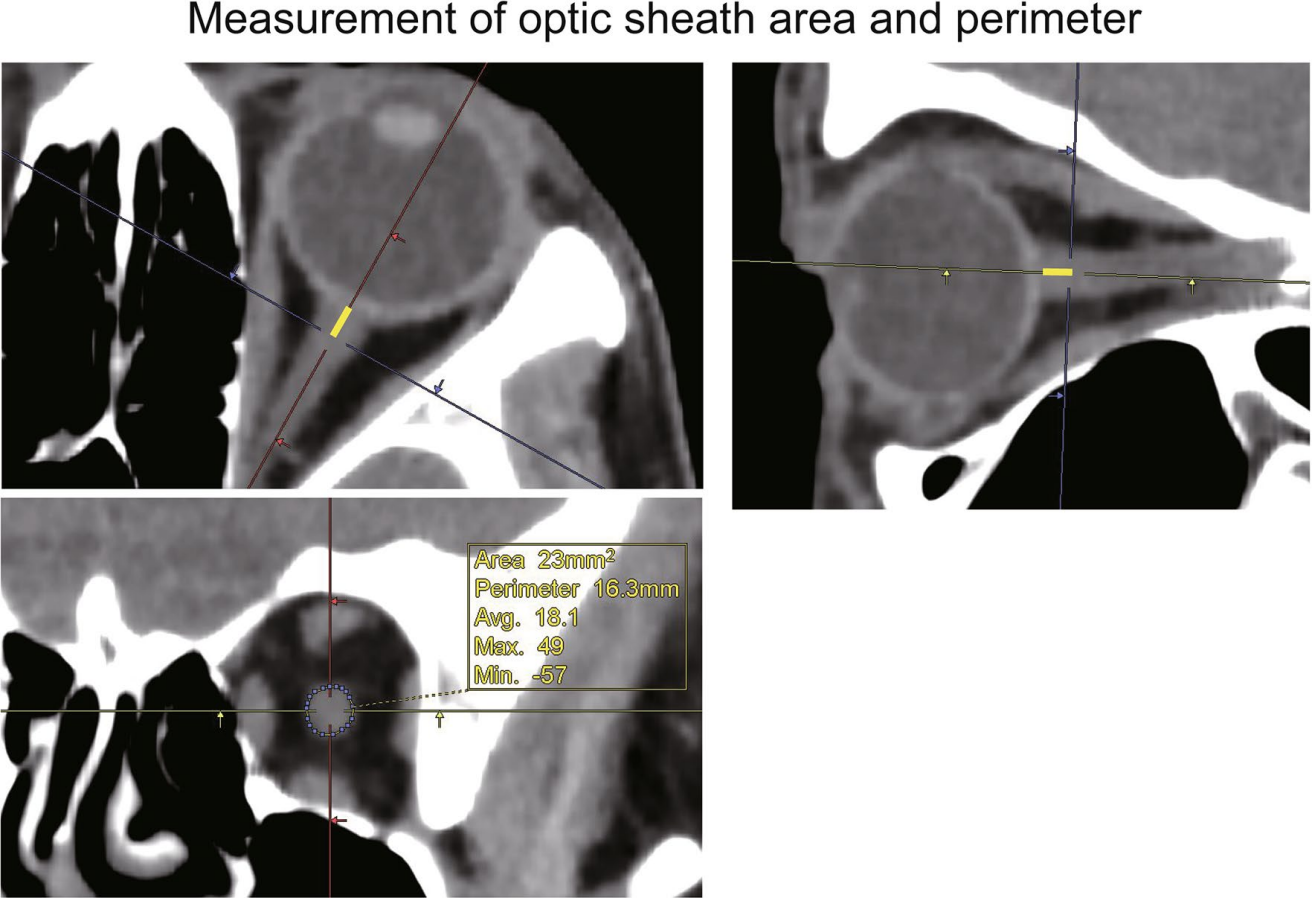
The optic nerve sheath area and perimeter (ONSA and ONSP, respectively) were measured 3 mm behind (yellow line) the eyeglobe on an oblique coronal image.

Regarding the pineal body shift, we measured the distance of the ventral/dorsal and cranial/caudal directions shifted by the pineal gland in the sitting position relative to its position in the supine position by using superimposed sagittal images of the supine and sitting positions. The shift value was positive for the cranial and ventral directions and negative for the caudal and dorsal directions. Regarding the length of the pituitary stalk, we measured the distance from the tip of the infundibular recess to the junction of the pituitary stalk and pituitary gland, along the course of the pituitary stalk, on a sagittal image (Fig. 2). Regarding the distance between the cerebellar tonsil and a reference line; on a sagittal image, the distance was positive above the BO line and negative below the BO line (Fig. 3). The ONSA and ONSP were measured 3 mm behind the eye globe on an oblique coronal image (Fig. 4). The region of the lateral ventricle was manually segmented in each CT slice. The intracranial volume was automatically segmented by using volumetric tool in the workstation.

All measurements were performed in a blinded and randomized manner. In all 32 cases, the first measurement was performed by a general radiologist with 5 years of experience. To maintain the intra-observer agreement, the second measurement was performed by the same reader 3 months after the first reading. To assess for interobserver agreement, 16 cases were evaluated by a neurosurgeon with 6 years of experience. The inter- and intra-observer agreements were assessed by measuring the intraclass correlation coefficients.

### Visual assessment of images

The radiologist and neurosurgeon achieved consensus on the obtained images, based on visual assessment of supine and sitting images. We evaluated shape changes in the CSF space between supine and sitting positions by observing them at high convexity, the sylvian fissure, supravermian cistern, and prepontine cistern (SI 2 for high convexity and the sylvian fissure, and SI 3 for supravermian cistern and prepontine cistern).

### Statistical analysis

Data are presented as mean ± standard deviation. A sample *t* test was used to assess the pineal body shift. Paired *t* tests were used to analyse changes the following parameters between the supine and sitting positions: length of the pituitary stalk, distance between the cerebellar tonsil and BO line, ONSA, ONSP, lateral ventricular volume, and intracranial volume.

The associations between the CT parameters and participants’ characteristics were evaluated with an analysis of covariance (ANCOVA).To ensure that the data met the assumptions underlying the statistical tests, the Shapiro–Wilk test of normality was used. The significance level for all tests was 5% (two-sided). All data were analysed using a commercially available software program (JMP; version 15, SAS, Cary, North Carolina, USA).

## Results

### Characteristics of the subject population

The age, body weight, body height, and body mass index of the male subjects were 48.3 ± 13.0 years (range 30–68 years), 65.9 ± 10.8 kg, 168.1 ± 6.3 cm, and 23.3 ± 3.36 kg/m^2^, respectively. The same parameters of the female subjects were 48.4 ± 10.2 years (range 33–63 years), 54.7 ± 7.50 kg, 158.6 ± 6.0 cm, and 21.7 ± 2.38 kg/m^2^, respectively (Table 1).

### Scan time, length, and effective dose for each position

The scan time was 4.4 ± 0.1 s for 171.1 ± 5.8 mm scans in the supine and sitting positions.

The effective dose estimate for the head was 1.95 ± 0.14 mSv in the supine position and 1.51 ± 0.24 mSv in the sitting position.

### Changes in intracranial structure between supine and sitting positions

The pineal body shifted 0.68 ± 0.27 mm in the ventral direction and 0.76 ± 0.24 mm in the caudal direction in the sitting position (all *P* < 0.001). The intra- and inter-observer agreements were 0.965 and 0.959, respectively, for the ventral/dorsal shift, and 0.966 and 0.958 for the cranial/caudal shift (Table 2).

**Table 2:** All parameters are described in the supine and sitting positions. In the sitting position, the pineal body shifted ventrally and caudally, cerebellar tonsil descended, and the optic nerve sheath area, perimeter, and lateral ventricular volume decreased significantly.

Changes in intracranial structure between the supine and sitting positions *n* = 32 (16 males and 16 females)					
Shift of the pineal body (mm)	In the ventral direction	*P*-value	Intra-observer agreements	Inter-observer agreements	
	0.68 ± 0.27	< 0.001	0.965	0.959	

Changes in intracranial structure between the supine and sitting positions *n* = 32 (16 males and 16 females)					
Shift of the pineal body (mm)	In the caudal direction	*P*-value	Intra-observer agreements	Inter-observer agreements	
	0.76 ± 0.24	< 0.001	0.966	0.958	

Changes in intracranial structure between the supine and sitting positions *n* = 32 (16 males and 16 females)					
	Supine	Sitting	*P-*value	Intra-observer agreements while supine/sitting	Inter-observer agreements while supine/sitting
Length of the pituitary stalk (mm)	4.29 ± 1.15	3.06 ± 1.07	< 0.001	0.941/0.860	0.821/0.849
Distance between the cerebellar tonsil and BO line (mm)	4.77 ± 3.79	2.67 ± 3.77	< 0.001	0.994/0.995	0.980/0.991
Right optic nerve sheath area (mm^2^)	26.20 ± 4.57	22.20 ± 4.17	< 0.001	0.950/0.945	0.912/0.931
Left optic nerve sheath area (mm^2^)	26.46 ± 5.78	22.30 ± 4.74	< 0.001	0.972/0.969	0.981/0.964
Right optic nerve sheath perimeter (mm)	17.15 ± 1.58	15.68 ± 1.48	< 0.001	0.948/0.941	0.940/0.935
Left optic nerve sheath perimeter (mm)	17.13 ± 1.83	15.71 ± 1.75	< 0.001	0.978/0.969	0.975/0.943
Lateral ventricular volume (ml)	16.54 ± 7.19	15.73 ± 6.97	< 0.001	0.980/0.977	0.971/0.987

*BO line*: basion-to-opthision line.

**Figure 5:**
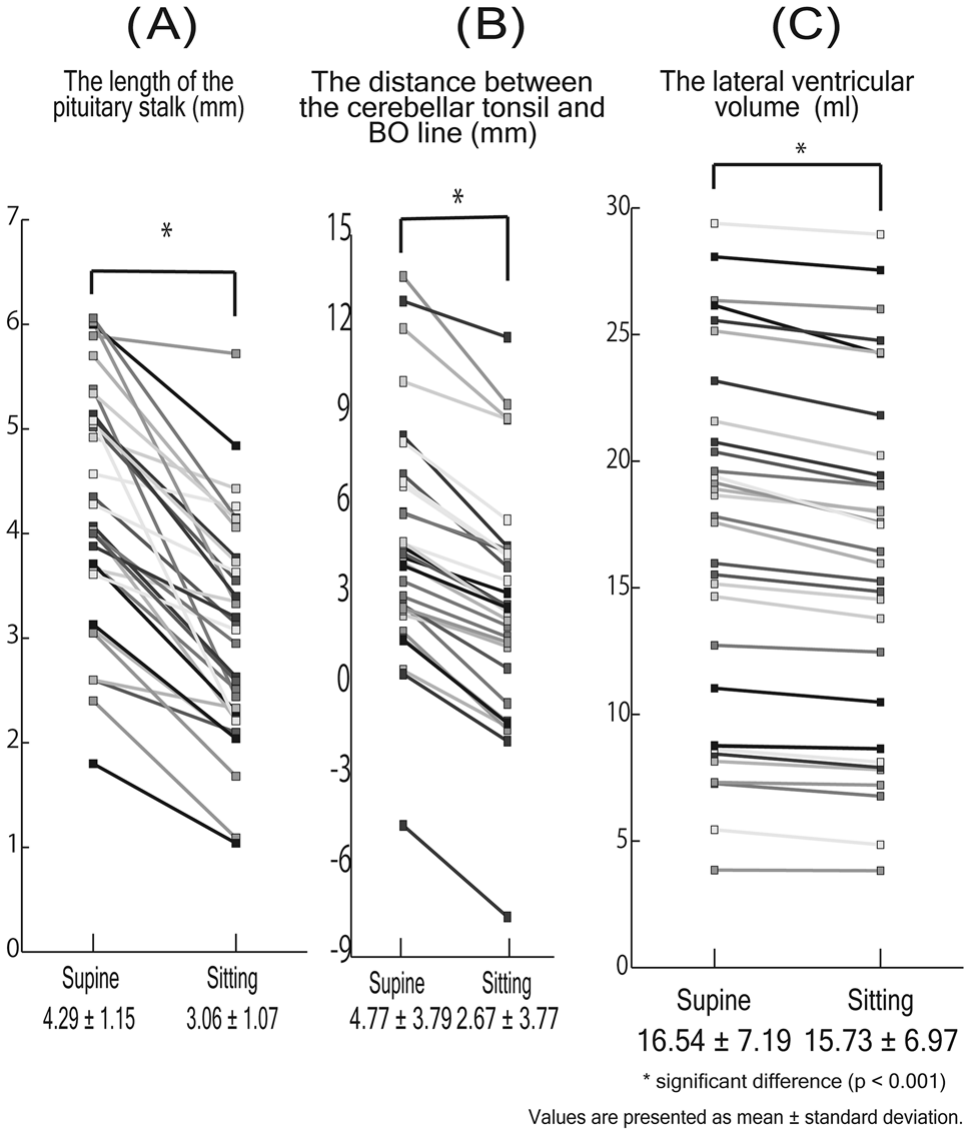
(**A**–**C**) The figure shows the distance between the cerebellar tonsil and BO line (left), the length of the pituitary stalk (middle), the lateral ventricular volume (right) in supine and sitting position. These parameters in sitting position was less than that in supine position significantly (p < 0.001).

The pituitary stalk shortened by 1.23 ± 0.71 mm in the sitting position (*P* < 0.001). The intra- and inter-observer agreements for the length of pituitary stalk were 0.941 and 0.821 in the supine position and 0.860 and 0.849 in the sitting position, respectively (Table 2; Fig. 5A).

The distance between the cerebellar tonsil and the basion to opisthion (BO) line was 4.77 ± 3.79 mm in the supine position and 2.67 ± 3.77 mm in the sitting position. Thus, this distance decreased by 2.10 ± 0.86 mm in the sitting position (*P* < 0.001). The intra- and the inter-observer agreements were 0.994 and 0.980, respectively, in the supine position, while those of the sitting position were 0.995 and 0.991, respectively (Table 2; Fig. 5B).

**Figure 6:**
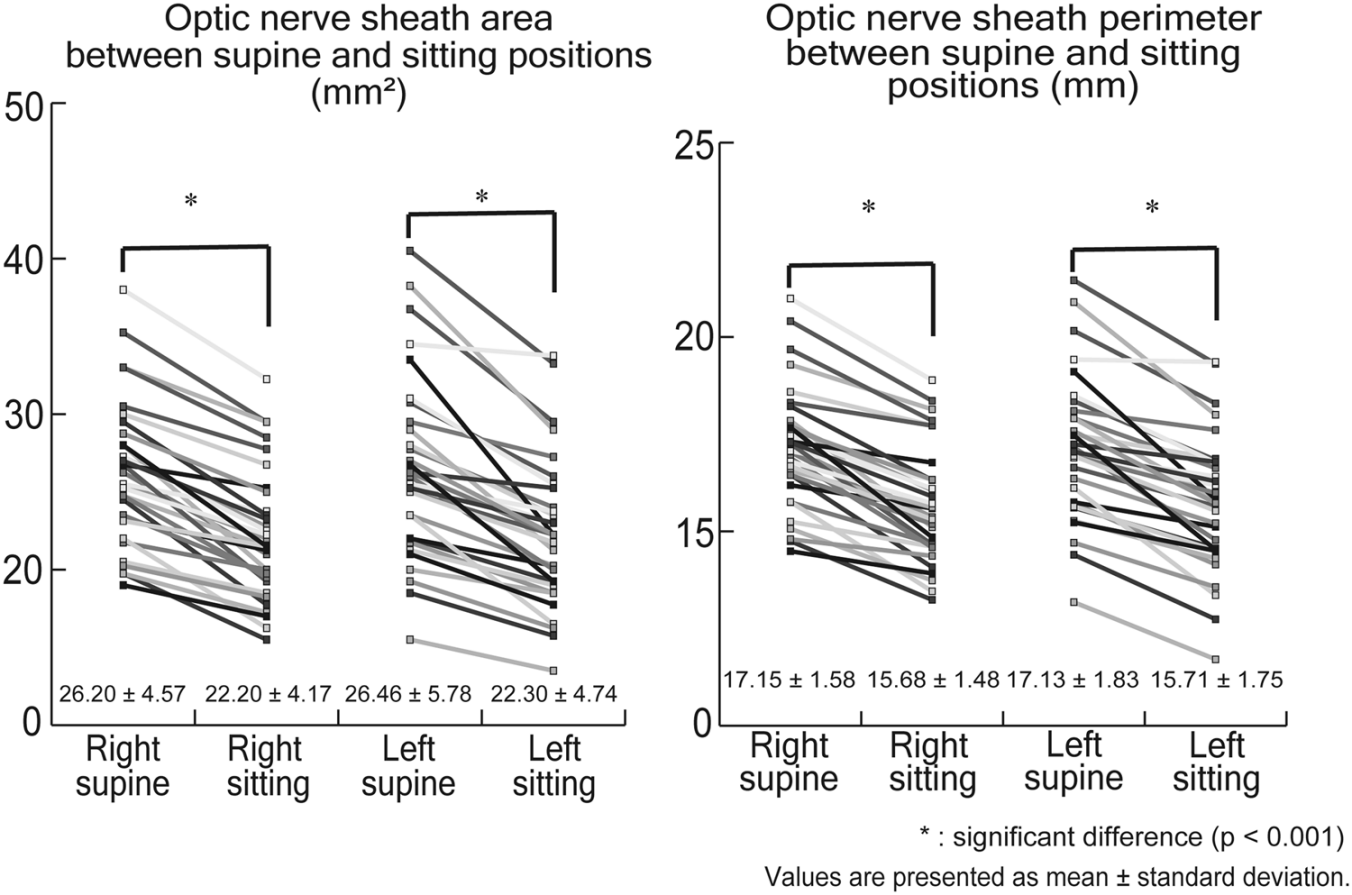
The figure shows ONSA and ONSP in supine and sitting position. Both ONSA and ONSP decreased in sitting position compared with supine position significantly (p < 0.001).

The right optic nerve sheath area (ONSA) was 26.20 ± 4.57 mm^2^ in the supine position and 22.20 ± 4.17 mm^2^ in the sitting position (*P* < 0.001). The left ONSA was 26.46 ± 5.78 mm^2^ in the supine position and 22.30 ± 4.74 mm^2^ in the sitting position (*P* < 0.001). Thus, the right ONSA decreased by 15.21 ± 6.54% (4.00 ± 1.83 mm^2^) and the left ONSA decreased by 15.30 ± 7.37% (4.16 ± 2.55 mm^2^) in the sitting position (each *P* < 0.001). The intra- and the inter-observer agreements for the right ONSA were 0.950 and 0.912 in the supine position and 0.945 and 0.931 in the sitting position, respectively. We did not find any significant differences in the ONSA between the two eyes in the supine (P = 0.65) and sitting positions (P = 0.82). Similarly, there were no significant differences in the ONSP between the two eyes in the supine (P = 0.91) and sitting positions (P = 0.82). The intra- and the inter-observer agreements for the left ONSA were 0.972 and 0.981 in the supine position and 0.969 and 0.964 in the sitting position, respectively (Table 2; Fig. 6).

The right optic nerve sheath perimeter (ONSP) was 17.15 ± 1.58 mm in the supine position and 15.68 ± 1.48 mm in the sitting position, and the left ONSP was 17.13 ± 1.83 mm in the supine position and 15.71 ± 1.75 mm in the sitting position (*P* < 0.001). The right ONSP decreased by 8.52 ± 3.91% (1.47 ± 0.69 mm) and the left ONSP decreased by 8.20 ± 4.38% (1.41 ± 0.81 mm) in the sitting position (each *P* < 0.001). The intra-observer and the inter-observer agreement for the right ONSP were 0.948 and 0.940 in the supine position, and 0.941 and 0.935 in the sitting position, respectively. The intra- and the inter-observer agreement for the left ONSP were 0.978 and 0.975 in the supine position, and 0.969 and 0.943 in the sitting position, respectively (Table 2; Fig. 6).

The lateral ventricular volume was 16.54 ± 7.19 ml in the supine position and 15.73 ± 6.97 ml in the sitting position. The lateral ventricular volume decreased by 5.07 ± 3.24% (0.81 ± 0.57 ml) in the sitting position (each *P* < 0.001). The intra- and the inter-observer agreement for the lateral ventricular volume were 0.980 and 0.971 in the supine position, and 0.977 and 0.987 in the sitting position, respectively (Table 2; Fig. 5C). The intracranial volume was 1410.0 ± 142.3 ml in the supine position and 1410.0 ± 142.3 ml in the sitting position. There were no significant differences in the intracranial volume between the two positions (P = 0.22).

### Analysis of covariance (ANCOVA) between the CT parameters and participants’ characteristics

The CT parameters that changed with the subjects’ posture showed an inter-subject variance; therefore, we additionally assessed our results using ANCOVA. In this model, the factors were: posture (supine or sitting position), age, gender, body height, and intracranial volume, taking into account the correlation among variables (e.g., height, weight, and body mass index).

The ANCOVA analysis demonstrated the following results: the pineal body shift in the cranial-caudal and ventral–dorsal directions was independently associated with posture (P < 0.0001) and gender (P = 0.0121) after adjusting for other clinical variables; distance between the cerebellar tonsil and BO line was independently associated with posture (P = 0.0181), gender (P = 0.0006), and body height (P = 0.0057); lateral ventricular volume was independently associated with age (P = 0.0005) and intracranial volume (P = 0.0106); bilateral ONSA and ONSP were associated with posture (P = 0.0005, 0.0003, 0.0026, and 0.0026, respectively); length of the pituitary stalk was independently associated with posture (P < 0.0001) and gender (P = 0.0464) (Supplementary Tables 1–9).

### Visual assessment of images

Based on their visual assessment, the two doctors achieved a consensus on the images.

In the sitting position, there was expanding of CSF spaces at high convexity in 22 of 32 subjects (69%) and no apparent change in the remaining 10 subjects as compared to that of the subjects in the supine position. The CSF spaces at the sylvian fissure were narrowed in 26 of 32 subjects (81%) and showed no apparent change in the remaining 6 subjects. The supravermian cistern was widened in 30 of 32 subjects (94%) and showed no apparent change in the remaining 2 subjects, while the prepontine cistern was narrowed in all subjects (100%).

## Discussion

To the best of our knowledge, this study is the first to elucidate changes in the intracranial structure due to posture and use upright CT images for the same. This study proved that the intracranial structure moves in accordance with postural change, which thus far, has remained undetected in previous vertical MRI studies owing to the thicker slice sections of magnetic resonance images (upright CT: 0.5 mm and vertical MRI: 4.0–5.0 mm)^1,2,6^. We revealed there were significant changes in the shift of the pineal body and the cerebellar tonsil position, evident shortening of the pituitary stalk, changes in the area and perimeter of the optic nerve sheath, and a decreased lateral ventricular volume. The pineal body may have shifted due to a shortened pituitary stalk, which occured due to the movement of the cerebrum and hypothalamus. We also visualized shape changes in the CSF space in the sitting and supine positions.

Previous literature describes that, when standing up, gravity displaces the blood and fluid towards the feet, thereby introducing a hydrostatic pressure gradient and reducing pressure in the cranial direction^9^. Furthermore, studies using upright MRI found that the hydrostatic CSF pressure would be lower at the upper cervical level than at the central cranial level^10^. The CSF space includes the ventricles and the cerebral and spinal subarachnoid spaces. The specific gravity of normal human CSF ranges from 1.0063 to 1.0075 and that of brain tissue ranges from 1.03 to 1.26^11,12^. Thus, the brain tissue appears to have a greater specific gravity than CSF. We believed that a change in posture would cause the hydrostatic pressure gradient to alter the distribution of CSF, and the volume and shape of CSF space would change. Since the specific gravity of the brain tissue is greater than that of the CSF, we concluded that the intracranial structure would be affected by gravity more in the sitting position than in the supine position and would descend consequently.

A previous study reported the effects of the time of day on brain tissue and CSF volume, where it was found that CSF volumes were increased in the evening as compared to the morning^13^. We performed all the CT examinations in the evening. The increased hydrostatic gradient due to the increased CSF in the evening may have also helped us detect the shift in intracranial structure due to postural changes.

According to Davson’s equation for absorption of CSF, an expression for baseline intracranial pressure (ICP) in the supine position was previously derived^14^:

$$ ICP \, = \, R_{out} \cdot \;I_{formation} + \, P_{d} $$

where R_out_ is the resistance to outflow of CSF, I_formation_ is the formation rate of CSF, and P_d_ is the pressure in the dural venous sinuses, assuming that R_out_ and I_formation_ are unaffected by body position^14^. This previous study evaluated postural effects on intracranial pressure and reported that higher raised tilt angles of the upper body resulted in decrease in the ICP. Another study that discussed the relationships between dural sinus pressure and posture reported that the confluens sinuum pressure in the sitting position decreased by − 12.7 ± 3.0 cm H_2_O (mean ± SD) as compared with the supine position^15^. Our subjects showed decreased lateral ventricular volume while seated; therefore, the change in CSF distribution may correlate with the previously established ICP decrease in the sitting position. Further study regarding the relationship among CSF outflow resistance/formation rate, ICP, and posture would be desired.

Recently, intracranial structural changes that were similar to our findings were reported in astronauts. One study found that the lateral ventricular volume of astronauts before and after spaceflight increased by 13.3 ± 1.9%^16^. The state of the intracranial structure after spaceflight appears to correspond to the intracranial findings in our supinely positioned subjects, where gravity did not affect their posture.

The above-mentioned study also found that even 7 months after their return from space, the astronauts’ ventricular volumes were enlarged as compared with their pre-flight volumes^16^. This is because microgravity acted chronically due to the long duration of the space mission (169 ± 24 days)^16^. In our study, lateral ventricular volume increased marginally by 5.07 ± 3.24% in the supine position as compared with the sitting position; this reflects the acute effect of gravity on the intracranial structure because we acquired CT images immediately after the positional change.

Another study reported that post-space flight sagittal MRIs of astronauts show an uplifting of the optic chiasm; this is assumed to occur because the optic nerve is pulled rearward due to the brain’s upward shift and its rotation around the edge of the cerebellar tentorium during space flight^4^. This rearward shift of the optic nerve may cause the optic nerve sheath to expand and bend, resulting in changes in the nerve sheath diameter because the periosteum is connected to the dura of the sheath at the orbit^4^. Therefore, since the diameter of the optic nerve sheath was increased, we assumed that ONSA and ONSP would increase as well.

These two studies that reported that the intracranial structure shifted after spaceflight are important since it has been recognized that the intracranial structure is stationary based on a vertical MRI study. Our study showed that change in the intracranial structure is also seen even as an acute effect of postural change on Earth.

Intracranial structural movement could play an important role in disease. One previous study of CSF leakage and CSF shunt overflow described that the consequence of CSF volume depletion was descent of the brain (i.e., a “sinking brain” or “sagging brain”), which would be probably even more pronounced and more frequently documented if the patient is upright rather than supine, a position in which patients are typically less symptomatic or asymptomatic^17^. In this study, we showed that the shift of the intracranial structure was observed even in healthy subjects. Our data of the amount of variation in the intracranial structure caused by postural changes in healthy subjects are important for determining the definition of an abnormal brain position.

In the future, upright CT can be applied to evaluate structural diseases of the brain, such as sinking flap syndrome following decompressive craniotomy. Atmospheric pressure has been implicated as the cause of this condition^18^. Because of removal of the skull in such cases, changes in the intracranial structure due to posture are expected to be more pronounced than in healthy individuals.

Our study had several limitations. First, our study include a very small number of included subjects and its single-institution design. Second, we conducted only unenhanced CT scans. Therefore, it was impossible to measure the venous structures of the brain precisely and we were unable to discuss the relationship between venous structures and postural changes.

In conclusion, we showed that the intracranial structure of healthy subjects shifts and volume of the ventricles changes even during short-term postural changes on Earth. Our data of healthy volunteers are important for determining the abnormal brain position in the diagnosis of intracranial hypotension.

## Supplementary Information

Supplementary Figure.

Supplementary Video 1.

Supplementary Video 2.

Supplementary Tables.

## Data Availability

The datasets generated during and/or analysed during the current study are available from the corresponding author on reasonable request.
