# Supplementary figures and images for: Effect of gravity on brain structure as indicated on upright computed tomography
Source: Sci Rep. 2021 Jan 11;11:392. doi: 10.1038/s41598-020-79695-z (PMC7801697; doi:10.1038/s41598-020-79695-z)

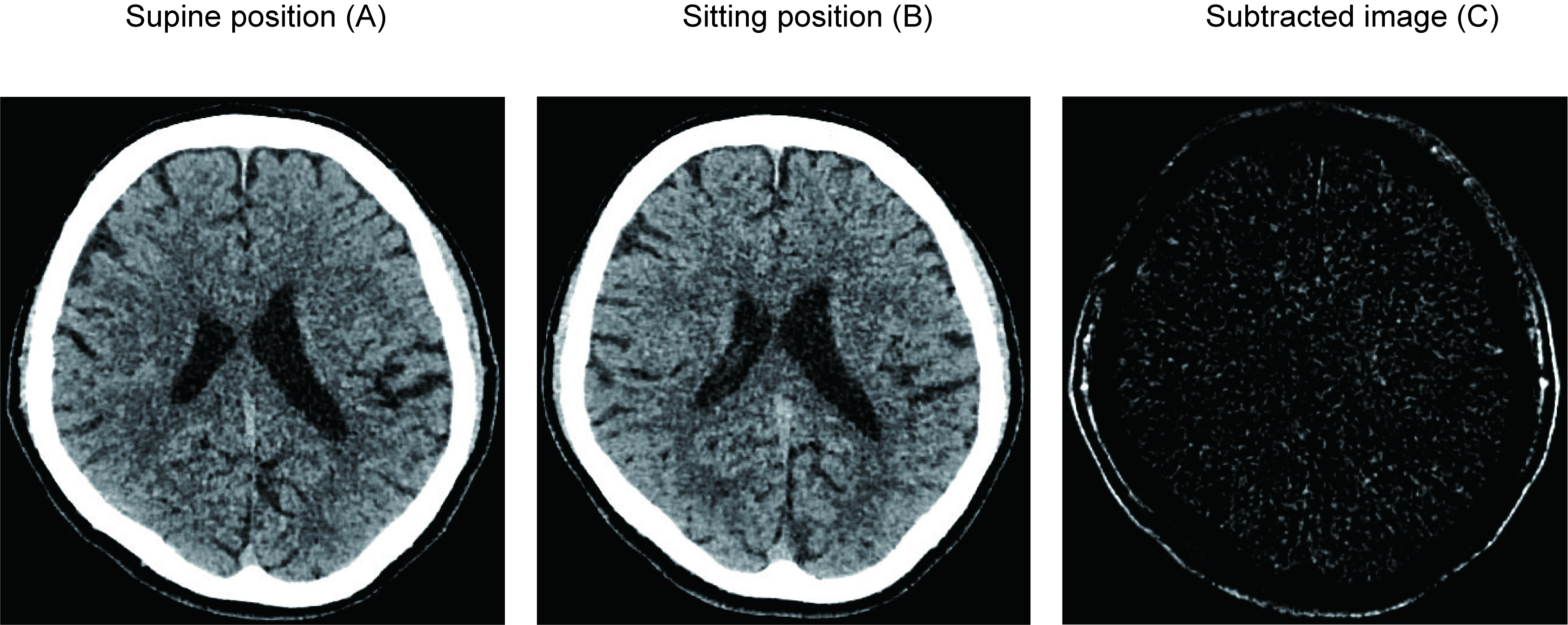

Supplement: Supplementary file 1 — Supplementary Figure. [file 41598_2020_79695_MOESM1_ESM.tif]
