# Supplementary material for: Effect of gravity on brain structure as indicated on upright computed tomography
Source: Sci Rep. 2021 Jan 11;11:392. doi: 10.1038/s41598-020-79695-z (PMC7801697; doi:10.1038/s41598-020-79695-z)
Supplement: Supplementary file 4 — Supplementary Tables. [file 41598_2020_79695_MOESM4_ESM.docx]

**Supplementary Tables 1-9**

The following tables describe the ANCOVA model between the computed tomography parameters and the participants’ characteristics.

(**Supplementary Tables 1**)

・Shift of the pineal body in the cranial-caudal direction.

| **Variable** | **Coefﬁcient^a^** | **Standard Error** | ***P-*value^b^** |
| --- | --- | --- | --- |
| Intercept | 0.9882659 | 0.605128 | 0.1079 |
| Supine vs. Sitting (Supine position) | 0.3813375 | 0.019813 | <.0001* |
| Age | -0.000831 | 0.001949 | 0.6712 |
| Gender (Male) | 0.0923104 | 0.027821 | 0.0016* |
| Body height | -0.00467 | 0.003958 | 0.2428 |
| Intracranial volume | -0.000402 | 0.000213 | 0.0639 |

(**Supplementary Tables 2**)

・Shift of the pineal body in the ventral-dorsal direction.

| **Variable** | **Coefﬁcient^a^** | **Standard Error** | ***P-*value^b^** |
| --- | --- | --- | --- |
| Intercept | -0.942826 | 0.710786 | 0.1899 |
| Supine vs. Sitting (Supine position) | -0.337754 | 0.023273 | <.0001* |
| Age | -0.001653 | 0.002289 | 0.4732 |
| Gender (Male) | -0.084645 | 0.032678 | 0.0121* |
| Body height | 0.0044343 | 0.004649 | 0.3442 |
| Intracranial volume | 0.0004513 | 0.00025 | 0.0761 |

(**Supplementary Tables 3**)

・Distance between the cerebellar tonsil and the basion-to-opisthion (BO) line.

| **Variable** | **Coefﬁcient^a^** | **Standard Error** | ***P*-value^b^** |
| --- | --- | --- | --- |
| Intercept | 44.548423 | 13.1646 | 0.0013* |
| Supine vs. Sitting (Supine position) | 1.0488368 | 0.43104 | 0.0181* |
| Age | -0.039745 | 0.042397 | 0.3524 |
| Gender (Male) | 2.1848008 | 0.605241 | 0.0006* |
| Body height | -0.247128 | 0.08611 | 0.0057* |
| Intracranial volume | 0.0010371 | 0.004627 | 0.8235 |

**(Supplementary Tables 4)**

・Lateral ventricular volume

| **Variable** | **Coefﬁcient^a^** | **Standard Error** | ***P*-value^b^** |
| --- | --- | --- | --- |
| Intercept | -43.91697 | 21.5835 | 0.0465* |
| Supine vs. Sitting (Supine position) | 0.3903108 | 0.706695 | 0.5829 |
| Age | 0.2582035 | 0.069511 | 0.0005* |
| Gender (Male) | -0.428724 | 0.992299 | 0.6673 |
| Body height | 0.118287 | 0.141178 | 0.4055 |
| Intracranial volume | 0.020035 | 0.007587 | 0.0106* |

(**Supplementary Tables 5**)

・ Right ONSA

| **Variable** | **Coefﬁcient^a^** | **Standard Error** | ***P*-value^b^** |
| --- | --- | --- | --- |
| Intercept | 5.182049 | 16.50324 | 0.7546 |
| Supine vs. Sitting (Supine position) | 2.0023126 | 0.540355 | 0.0005* |
| Age | -0.064129 | 0.053149 | 0.2325 |
| Gender (Male) | -0.432212 | 0.758735 | 0.5711 |
| Body height | 0.1422432 | 0.107948 | 0.1928 |
| Intracranial volume | -0.00079 | 0.005801 | 0.8921 |

(**Supplementary Tables 6**)

・Right ONSP

| **Variable** | **Coefﬁcient^a^** | **Standard Error** | ***P*-value^b^** |
| --- | --- | --- | --- |
| Intercept | 10.664208 | 5.782776 | 0.0703 |
| Supine vs. Sitting (Supine position) | 0.735177 | 0.189342 | 0.0003* |
| Age | -0.020017 | 0.018624 | 0.2869 |
| Gender (Male) | -0.113041 | 0.265863 | 0.6723 |
| Body height | 0.0419697 | 0.037825 | 0.2718 |
| Intracranial volume | -9.533e-5 | 0.002033 | 0.9628 |

(**Supplementary Tables 7**)

・Left ONSA

| **Variable** | **Coefﬁcient^a^** | **Standard Error** | ***P*-value^b^** |
| --- | --- | --- | --- |
| Intercept | 6.0346474 | 20.15305 | 0.7657 |
| Supine vs. Sitting (Supine position) | 2.0804714 | 0.659859 | 0.0026* |
| Age | -0.078945 | 0.064904 | 0.2288 |
| Gender (Male) | -1.063665 | 0.926535 | 0.2557 |
| Body height | 0.0738549 | 0.131821 | 0.5775 |
| Intracranial volume | 0.007166 | 0.007084 | 0.3159 |

(**Supplementary Tables 8**)

・Left ONSP

| **Variable** | **Coefﬁcient^a^** | **Standard Error** | ***P*-value^b^** |
| --- | --- | --- | --- |
| Intercept | 10.222688 | 6.852369 | 0.1412 |
| Supine vs. Sitting (Supine position) | 0.7057645 | 0.224363 | 0.0026* |
| Age | -0.024383 | 0.022068 | 0.2738 |
| Gender (Male) | -0.316837 | 0.315037 | 0.3187 |
| Body height | 0.0259447 | 0.044821 | 0.5649 |
| Intracranial volume | 0.0022303 | 0.002409 | 0.3583 |

(**Supplementary Tables 9**)

・Pituitary stalk

| **Variable** | **Coefﬁcient^a^** | **Standard Error** | ***P*-value^b^** |
| --- | --- | --- | --- |
| Intercept | 9.8385219 | 4.083224 | 0.0192* |
| Supine vs. Sitting (Supine position) | 0.6137525 | 0.133694 | <.0001* |
| Age | 0.0194725 | 0.01315 | 0.1441 |
| Gender (Male) | 0.3821327 | 0.187726 | 0.0464* |
| Body height | -0.037216 | 0.026708 | 0.1688 |
| Intracranial volume | -0.000729 | 0.001435 | 0.6134 |

^a^Coefficient indicates an increase in the dependent variable for each one-unit increase in an independent variable.

^b^P-values were calculated using multiple linear regression analysis.

*indicates P < 0.05.
